# Bardet-Biedl Syndrome: A Rare Case From Ophthalmology Perspective

**DOI:** 10.7759/cureus.29912

**Published:** 2022-10-04

**Authors:** Mustafa Alhamoud, Ghadah Alnosair, Hassan Alhashim

**Affiliations:** 1 Ophthalmology, King Fahd Hospital of the University, Dammam, SAU; 2 Pediatric Ophthalmology and Strabismus, Dammam Medical Complex, Dammam, SAU; 3 Ophthalmology, Imam Abdulrahman Bin Faisal University, Dammam, SAU

**Keywords:** laurence-moon-bardet-biedl syndrome, retinitis pigmentosa, polydactyly, relatives’ marriage, consanguineous marriage, bardet-biedl syndrome

## Abstract

Bardet-Biedl syndrome (BBS) is a rare multisystem disease, with autosomal recessive inheritance and genetic heterogeneity characterized by post-axial polydactyl, cone-rods dystrophy, and central obesity. BBS involves many organs in the body with variable complications and the life span of affected individuals. Clinical confirmation of the disorder can be done using a revised criterion that consists of primary or major features and secondary or minor features. Primary features of BBS include hypogonadism, polydactyly, obesity, retinitis pigmentosa, and learning disability. While ataxia, poor coordination, brachydactyly, diabetes mellitus, speech abnormalities, liver fibrosis, hearing loss, spasticity, and cardiovascular anomaly constitute the secondary features. In this study, we report a case of a five-year-old Saudi girl who presented with language delay, delay in milestones, progressive weight gain, excised polydactyly, and retinitis pigmentosa. An integrated medicine approach would substantially improve the quality of life of the affected individuals and their families by enhancing both physical and mental health.

## Introduction

Bardet-Biedl syndrome (BBS) is a rare multisystem disease, with autosomal recessive inheritance and genetic heterogeneity characterized by post-axial polydactyl, cone-rods dystrophy, and central obesity. On the other hand, there is another syndrome similar to BBS called Laurence-Moon syndrome (LMS) predominantly presented with spasticity and distal muscle weakness which is an autosomal recessive disease that has a complex systemic involvement too and is considered a major differential diagnosis that overlaps with BBS to produce a complicated and unfortunate clinical presentation called Laurence-Moon-Bardet-Biedl syndrome (LMBBS). Literature suggests that the two syndromes are distinct even if symptoms of the two syndromes overlap [1]. Differences between BBS and LMS in the clinical presentation are subtle but there are important clues that might be helpful to differentiate between them. Studies show that the difference between the two syndromes can be noticed in the core features as post-axial polydactyl and central obesity are mostly identified in BBS while spasticity and distal muscle weakness are more predominant in LMS which is a much rare syndrome [1]. Despite that, evidence also showed similar phenotypic features between the two syndromes, but it might have genotypic differences hence genetic analysis might help to reach an accurate final diagnosis [2].

BBS involves many organs in the body with variable complications and may shorten the life span of affected individuals [1]. Affected individuals suffer from systemic manifestations including neurodevelopmental abnormalities such as poor coordination and hypertonia in both upper and lower extremities. In addition to epilepsy and speech abnormalities, they suffer from behavioral and psychiatric abnormalities that include anxiety, mood disorders, and obsessive-compulsive behaviors. Hypogonadism which manifests as delayed onset of secondary sexual characteristics in both sexes is another important clinical feature that is diagnosed early in males because of small testes and micropenis, while in females it manifests as the hypoplastic uterus, septated vagina, vaginal atresia, and menstrual abnormalities. However, infertility is common in both sexes.

Another manifestation is central obesity which appears in the first year of life despite a normal birth weight. Additional digits in the hand or foot called postaxial polydactyl are a distinct manifestation found in a patient with BBS since birth. Comorbidities include chronic kidney disease, hypertension, and type 2 diabetes mellitus with chronic kidney disease that leads to end-stage renal disease being the major cause of morbidity and mortality [3]. Furthermore, clinical ophthalmic findings include constricted visual field, color vision abnormalities, poor visual acuity, and possibly complete blindness due to photoreceptors impairment with macular involvement in addition to retinitis pigmentosa and retinal dystrophy [4].

## Case presentation

**Figure 1:**
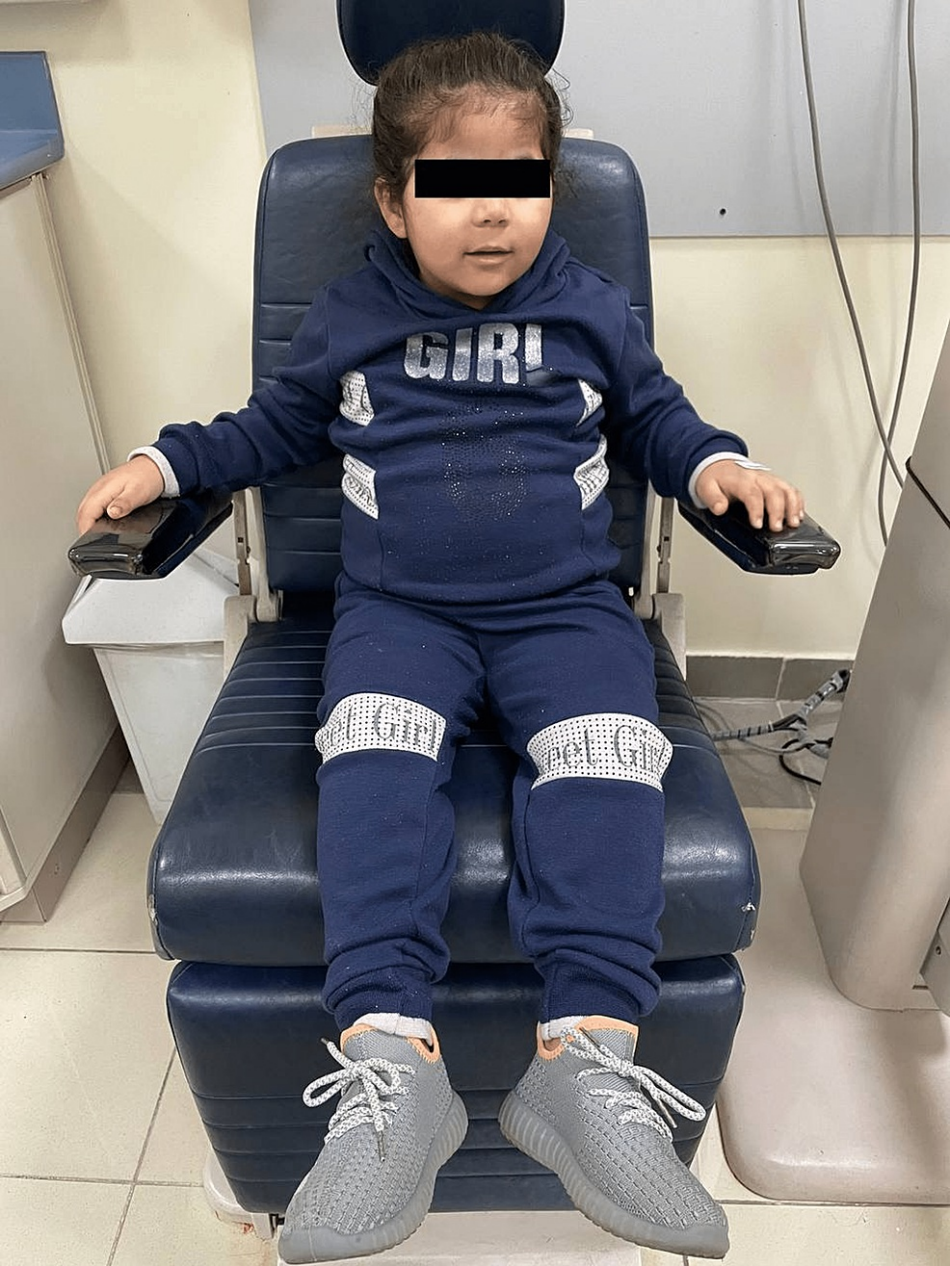
External picture of the patient showing central obesity with a moon-like face and some of the other above-mentioned dysmorphic features.

**Figure 2:**
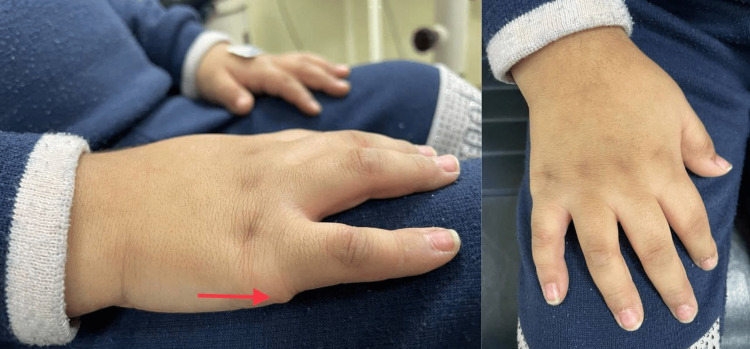
Scar in the right hand for previous polysyndactyly excision.

A five-year-old Saudi girl presented to the hospital with the main complaint of poor vision for two years, especially at night in the last two years. For her medical history, she was a product of a full-term uneventful pregnancy and a first baby in consanguineous marriage. Her mother mentioned that she is following up with the pediatric clinic for assessing her daughter’s difficulty in swallowing, language delay, delay in milestones, polyuria, and polydipsia with progressive weight gain. There was a positive family history of a similar condition, and the past surgical history reveals that she was diagnosed with right-hand polydactyly for which she did an excisional procedure before three years. Upon general physical examination, she had a subnormal mentality, central obesity, a moon-like face, and some dysmorphic features including microcephaly, narrow forehead, frontal bossing, low hairline, synophrys, depressed nasal bridge, upturned nostrils, long philtrum, micrognathia, small teeth, thick ears, and long eyelashes. As for the hand and foot examination, there was a scar on the right hand indicating post-axial polysyndactyly excision, spindle-shaped fingers, fifth finger clinodactyly, and two to three toe syndactyly (Figures 1, 2).

**Figure 3:**
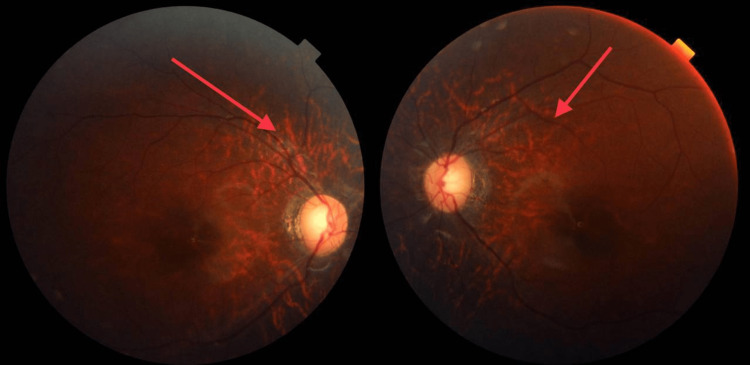
Tigroid fundus with pigmentary changes in their peripheral and mid-peripheral retina, mild attenuation of blood vessels with peripapillary atrophy.

Upon thorough ophthalmological assessment, the visual acuity was central steady and maintain in both eyes (OU), the external ocular movements were normal OU while the fundoscopic examination showed a tigroid fundus (Figure 3). Her cycloplegic refractive examination reveals myopic astigmatism (OD sphere: -3.75 cylinder -2.75 axis 180'; OS sphere: -2.50 cylinder -3.00 axis 10'). The patient did an electroretinogram (ERG) which shows severe photoceptor changes bilaterally in terms of diminished and delayed scotopic and photopic responses (rods are more affected than cons). Her basic lab workup including complete blood count (CBC), fasting blood sugar (FBS), thyroid-stimulating hormone (TSH), free T4, liver function test (LFT), and hemoglobin electrophoresis were within normal ranges, while renal function test (RFT) showed hyperkalemia (5.9 mmol/L) and elevated triglycerides level (2.84 mmol/L). The initial plan was to give her full corrective spectacles and follow-up after six months and request a genetic workup to identify any possible mutations consistent with BBS.

In the next follow-up, using next-generation sequencing (NGS) and whole exome sequencing (WES) genetic test, the report came back and the studied genes were ALMS1, ARL6, BBS1, BBS2, BBS4, BBS5, BBS7, BBS9, BBSI0, BBS12, CCDC28B, CEP290, IFT27, LZTFL1, MKKS, MKS1, SDCCAG8, TMEM67, TRIM32, TTC8, WDPCP. The result showed the variant of uncertain significance c.1553-1G>A p.? was detected in probable homozygosity in the BBS9 gene in chromosome 7 with an autosomal recessive pattern of inheritance.

Variants are reported for further clinical evaluation in the "potentially relevant variants" which means that this result might support a genetic etiology for the clinical presentation of our patient which concludes to diagnose our patient with Bardet-Biedl syndrome 9 (BBS9).

Thus, we planned to continue the follow-up in the pediatric ophthalmology clinic for optimizing her vision as much as possible, refer the patient to a tertiary hospital to follow-up with her systemic manifestations in an integrated pediatric clinic, test the parents to establish their status as carriers, and test affected relatives if available for the detected variant to evaluate its segregation and help to clarify its pathogenicity and eventual contribution to the disease development. Additionally, genetic counseling is recommended to decrease the risk of having the same condition in future pregnancies.

## Discussion

The incidence of BBS in North America and Europe varies between 1:140,000 to 1:160,000 live births. On the other hand, the incidence rate in the Arab world, particularly Kuwait, is much higher as it is 1:13,500 per live birth. This increase in incidence in the Arab world and the Middle East can be attributed to consanguineous marriage as it is quite common practice in that part of the world [4,5]. Even though the prevalence of BBS in Saudi Arabia is unknown, studies show that the rate of consanguinity in Saudi Arabia is quite high reaching up to 50% with 28.4% of them married to the first cousin, this might be a predictor of how common hereditary disorders are and especially BBS as studies show that 48% of affected patients were results of consanguinity as also seen in our patient [4,6].

Clinical confirmation of the disorder can be done using a revised criterion that consists of primary or major features and secondary or minor features. Primary features include hypogonadism, polydactyly, obesity, retinitis pigmentosa, and learning disability. While ataxia, poor coordination, brachydactyly, diabetes mellitus, speech abnormalities, liver fibrosis, hearing loss, spasticity, and cardiovascular anomaly constitute the secondary features [7]. According to the modified diagnostic criteria by Forsythe and Beales, four major features or three major features with two minor features are enough to establish the diagnosis [8]. For this report, our patient presented with polydactyly, obesity, and tigroid fundus which might be early changes of retinitis pigmentosa as primary features. For secondary features, our patient has established diabetes mellitus on treatment.

Since this is a rarely encountered disease, the ocular involvement in BBS is detected lately, and unfortunately, devastating consequences could be established, starting with nocturnal poor visual acuity which could be secondary to pigmentary retinopathy and macular affection and commonly ends up with a complete visual loss. Symptoms in BBS patients appear in the first 10 years of their life, with a chief complaint of poor night vision, nystagmus, photophobia, and blurring in central vision. This is the result of rod-cone photoreceptors dystrophy which is sometimes called atypical retinitis pigmentosa [4,9]. Our patient presented complaining of poor vision for two years, especially worsen at night.

Advances in the genetic field allowed scientists to identify specific mutations related to the development of BBS and those are BBS1, BBS2, ARL6/BBS3, MKKS/BBS6, BBS7, TTC8/BBS8, B1/BBS9, BBS10, TR1M32/BBS11 genes [1,10,11]. Hence, there are multiple phenotypes of BBS depending on the location of the mutated gene which differs in the severity of the disease and the majority of reported cases are linked to mutations found in BBS1 and BBS10 genes [8]. In our patient, genetic testing detects the variant of uncertain significance c.1553-1G>A p.? in probable homozygosity in the BBS9 gene in chromosome 7 with an autosomal recessive pattern of inheritance which confirms our clinical diagnosis of BBS9.

## Conclusions

Although BBS is considered a rare disease, it imposes a substantial burden on patients and their families for its multi-system involvement and disabling complications. A better understanding of this condition and its clinical manifestations will enable the health care providers to detect the disease in its beginning and hence, enhance their quality of life. Genetic counseling plays a significant role in raising community awareness about the potential genetic diseases associated with consanguineous marriage and screening the other members of the affected families, especially in our community. New modalities of treatment including gene therapy and targeted therapy considered promising hope in such diseases.
